# AggreBots: Configuring CiliaBots through guided, modular tissue aggregation

**DOI:** 10.1126/sciadv.adx4176

**Published:** 2025-09-26

**Authors:** Dhruv Bhattaram, Kian Golestan, Xuanshuo Zhang, Shihong Yang, Zhuowei Gong, Steven L. Brody, Amjad Horani, Victoria A. Webster-Wood, Amir Barati Farimani, Xi Ren

**Affiliations:** ^1^ Department of Biomedical Engineering, Carnegie Mellon University, Pittsburgh, PA, USA.; ^2^ Department of Medicine, Washington University School of Medicine, St. Louis, MO, USA.; ^3^ Department of Pediatrics, Washington University School of Medicine, St. Louis, MO, USA.; ^4^ Department of Cell Biology and Physiology, Washington University School of Medicine, St. Louis, MO, USA.; ^5^ Department of Mechanical Engineering, Carnegie Mellon University, Pittsburgh, PA, USA.

## Abstract

Ciliated biobots (CiliaBots) are engineered tissues capable of self-actuated propulsion via exterior motile cilia. While correlations have been observed between CiliaBot motility and morphology, direct control of morphological features to deliver desired motility outcomes remains unexplored. Here, we describe the engineering of aggregated CiliaBots (AggreBots) to augment control over CiliaBot structural parameters and, consequently, motility patterns through guided, modular aggregation of human airway epithelial spheroids [referred to as CiliaBot building blocks (CBBs)]. Multi-CBB aggregation generated rod-, triangle-, and diamond-shaped AggreBots, altering tissue geometry without sacrificing surface cilia density or inter-CBB boundary fidelity. The further introduction of *CCDC39*-mutated CBBs as cilia-inactive modules enabled the generation of hybrid AggreBots with precision modulation of active cilia distribution, further empowering alterations to motility patterns. Our results demonstrate the potential of AggreBots as living tissue propellers with morphological “levers” by which modifications to tissue motility can be theoretically planned and experimentally verified.

## Teaser

Multispheroid aggregation augments control over CiliaBot motility by configuring tissue morphology and active cilia distribution.

## INTRODUCTION

Stem cells, with their inherent ability to replicate and differentiate in response to a wide variety of intrinsic and extrinsic cues, have long been seen as the quintessential building block for manufacturing de novo biological constructs. In particular, the ability of stem cells to self-assemble can be strategically leveraged to give rise to by-design functional outcomes (*1*). This controlled functionality has led to stem cells being of interest in the growing field of biohybrid and organic robots, or biobots, where the ability of by-design function in living tissues is vital for delivering desired robot behavior (*2*–*7*). Biobots are living constructs capable of self-actuation whereby their designs, both the composing cells and their organization, can drive motility, the ability to move independently, powered by metabolic energy (*8*–*10*). The constituent cells within a biobot play a principal role in how the biobot actuates motility, with the most commonly investigated being muscle cells from cardiac or skeletal tissues, which produce forces through myofibril contraction (*4*, *11*–*14*). However, myofibrils do not represent the only possibility for a force-generating building block when designing a biobot.

Beyond muscles, another possibility for generating macroscopic motility with living tissues lies in motile cilia, hair-like cellular appendages of ~200 nm in diameter, ranging from 1 to 10 μm in length (*15*, *16*). Each of these cilia comprises a carefully arranged collection of microtubules to which dynein motor proteins are bound that together produce a force-generating “effective stroke” followed by a “recovery stroke.” Cilia can be found on the apical surface of several epithelium-enclosed tissue compartments in the human body, such as the respiratory airways, brain ventricles, middle ears, and fallopian tubes (*17*–*24*). Coordinated cycles of effective and recovery strokes at a frequency of 10 to 40 Hz from a large number of cilia, up to trillions in the lungs, generate macroscale motility in the form of metachronal waves that propel luminal substances, such as moving mucus in a cephalic direction along the respiratory tracts or transporting eggs through the fallopian tubes to the uterus (*17*, *18*, *23*, *24*). Looking back in evolution, motile cilia also represent an ancient mechanism for certain aquatic organisms to propel themselves through aqueous environments, from unicellular eukaryotic ciliates like *Paramecium* that have relied on cilia-powered propulsion for hundreds of millions of years to the multicellular Ctenophora family of marine invertebrates that swim the world’s oceans via their cilia, being the largest organisms to do so (up to 1.5 m in length) (*25*, *26*).

Not until recently has the leap to human cell–derived ciliated biobots (CiliaBots) been brought to reality. These human CiliaBots are three-dimensional (3D) organotypic mini-tissues (i.e., organoids) that are differentiated from stem cells of the human respiratory airways, thereby recapitulating the cellular composition of their tissue of origin, including the multiciliated cells (*1*, *10*, *27*). Prior work from us and others establishes that withdrawing extracellular matrix support from the culture environment is key to enabling the cilia-bearing apical epithelial surface to face the organoid’s exterior. Such apical-out tissue polarity allows the outward-facing cilia to drive macroscopic organoid motility, making them human CiliaBots. When embedded within a hydrogel substrate, these CiliaBots can generate stable motility in the form of 3D rotation, with the resulting activity being strongly linked to the beat frequency of constituent cilia (*27*). Intriguingly, comprehensive investigations of CiliaBot 2D locomotion when allowed to traverse a flat surface revealed a correlation between motility patterns and structural features such as tissue morphology and surface cilia coverage (*10*). But while these prior investigations provide valuable observations of spontaneously generated CiliaBot variations, a strategy for precisely controlling these structural parameters has yet to be established, presenting a challenge in developing CiliaBots with predefined structure and motility.

**Fig. 1.**
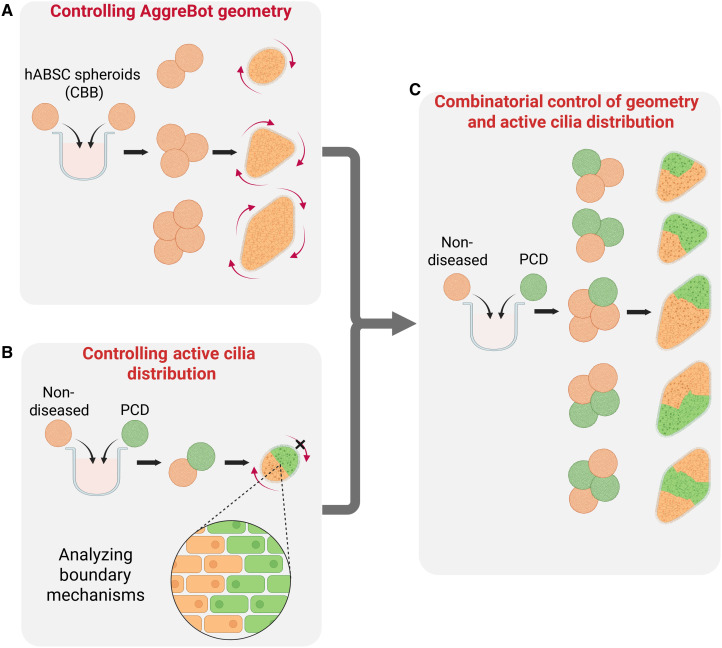
Schematic overview of AggreBot engineering. (**A**) Establishing geometric control of nascent AggreBots via spatiotemporally controlled aggregation of immature CBBs. (**B**) Establishing control of active cilia coverage and distribution in hybrid AggreBots through the introduction of PCD-derived CBBs (to designate domains of cilia inactivity), along with investigating maintenance of the boundary between constituent CBBs. (**C**) Using combinatorial control of AggreBot geometry and active cilia distribution to generate ever-increasing permutations of AggreBots, effecting additional motility patterns. Created in BioRender. Ren, X. (2025) https://BioRender.com/k07c325.

To address this need for manufacturing CiliaBots with predictable motility features by design, we present the concept and platform to engineer aggregated CiliaBots (AggreBots). These higher-order CiliaBots leverage spatiotemporally controlled placement and aggregation of individual CiliaBot building blocks (CBBs). Each CBB is a cluster of a defined number of human airway stem cells, which, on their own, can differentiate into simple, spherical CiliaBots of reproducible size and with homogeneous surface coverage of high-density motile cilia. Aggregation of these CBBs in a spatially directed manner allows for control over the resulting mature tissue geometry, which was used herein to generate several fundamental biobot designs, including rod-, triangle-, and diamond-shaped AggreBots. Additionally, we established a capability for control over the AggreBots’ active cilia coverage by introducing CBBs bearing nonfunctional cilia, leveraging airway stem cells with a genetic mutation responsible for primary ciliary dyskinesia (PCD) (*28*–*32*). The combination of controls over both tissue geometry and active cilia coverage and, by extension, functional cilia distribution allows for AggreBots to generate distinct and replicable phenotypes of 2D locomotion that have been herein quantified through their translational and rotational velocities. This analysis is further accompanied by a unique “path-and-extent” visualization method to transduce quantitative data into easily identifiable motility phenotypes (Fig. 1). Together, the research herein solidifies the AggreBot as a next-generation CiliaBot platform with the potential to support future applications in cell-based therapeutic delivery and ciliary theragnostics, due to the AggreBot platform’s cutting-edge design flexibility and reproducibility.

## RESULTS

### Assembly of geometrically configurable CiliaBots through modular aggregation of CBBs

Our previously established CiliaBot protocol showcased the ability of human airway basal stem cells (hABSCs) isolated from nondiseased (ND) donors and seeded into a U-bottom, cell-repellent microplate to cluster into spherical tissues in the absence of extracellular matrix support. These tissues could then further differentiate into CiliaBots with an apical-out epithelial polarity, where the exterior-facing motile cilia effectively propel tissue motility (*27*). However, this prior protocol did not allow for the production of tissue geometry beyond the default spherical shape. To address this, in this work, we hypothesized that altering CiliaBot tissue geometry could be achieved through spatially guided aggregation of individual spherical CiliaBot units, which could, by extension, have a controllable effect on CiliaBot motility. To explore this idea, we first attempted to merge a pair of fully differentiated CiliaBots to generate nonspherical geometries. However, this attempt failed because of interference from the constant cilia beating on the CiliaBots’ exterior surfaces that repelled one CiliaBot from the other, preventing stable contact (fig. S1 and movie S1).

**Fig. 2.**
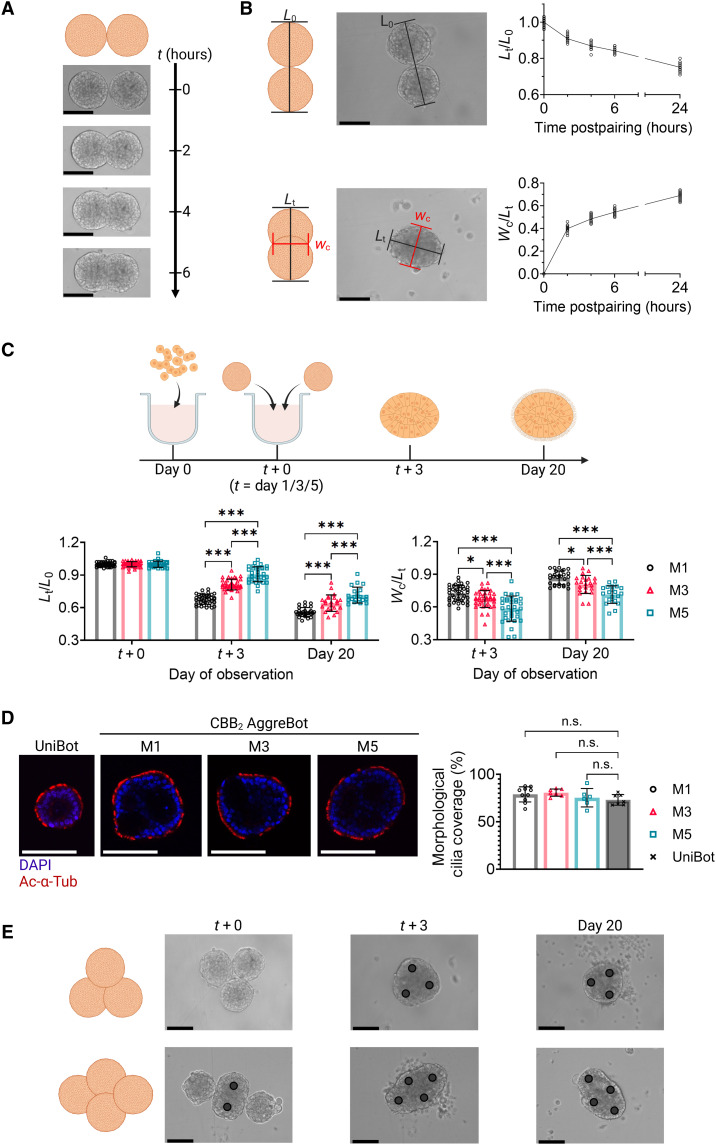
Characterization of CBB aggregation and resulting AggreBot morphology. (**A**) Time-series images of the aggregation behavior of two CBBs between 0 and 6 hours after pairing. (**B**) Depiction and quantification of normalized length (*L*_*t*_/*L*_*0*_) and width-length ratio (*W*_*c*_/*L*_*t*_) over the course of ${\text{CBB}}_{2}$ aggregation of 1-day-old CBBs between 0 and 24 hours after pairing, with accompanying schematic (*N* = 22). (**C**) Quantification of normalized length (*L*_*t*_/*L*_*0*_) and width-length ratio (*W*_*c*_/*L*_*t*_) of 2-CBB aggregation at CBB ages of 1, 3, and 5 days. Quantification was done immediately following CBB pairing (*N* = 112), 3 days following pairing (*N* = 109), and as the AggreBot approached tissue maturation at day 20 (*N* = 72), with accompanying schematic. (**D**) Example confocal slices of UniBots and ${\text{CBB}}_{2}$ AggreBots with immunostaining of Ac-α-Tub and DAPI, along with quantification of morphological cilia coverage (*N* = 30). (**E**) Time-series images of ${\text{CBB}}_{3}$ triangle-shaped and ${\text{CBB}}_{4}$ diamond-shaped AggreBot formation with images shown immediately following pairing with the last CBBs introduced, 3 days following pairing, and immediately before tissue maturation at day 20. Black circle markers overlaid on images to aid in the identification of constituent CBBs. All data represent means ± SD. *P < 0.05 and ***P < 0.001. n.s., not significant. Two-way ANOVA with Tukey’s multiple comparisons test in (C) and one-way ANOVA with Tukey’s multiple comparisons test in (D). Scale bar in all panels, 125 μm. Created in BioRender. Ren, X. (2025) https://BioRender.com/i02a255.

On the basis of this initial finding, we subsequently assessed the feasibility of aggregating epithelial spheroids before tissue maturation and cilia emergence. Seeding of a set number (500) of ND hABSCs on a cell-repellent, U-bottom microwell surface formed spheroids with homogeneous size distribution within 1 day of culture. These 1-day-old spheroids were then paired and combined in the same well, where the U-bottom well curvature brought the pairs into contact. Within as soon as 2 hours following initial pairing, adhesion and aggregation of the spheroid pairs were readily observed, compacting in combined tissue length and expanding in width of the contact surface as culture continued (Fig. 2A).

To quantify the extent of spheroid aggregation observed in the paired culture process, two morphological metrics were developed: (i) normalized length (*L*_*t*_/*L*_*0*_), defined as the length of the aggregate’s major axis at a given time (*L*_*t*_) as a ratio of the average initial pre-aggregation end-to-end lengths (*L*_*0*_) of the spheroid pairs; and (ii) width-length ratio (*W*_*c*_/*L*_*t*_), defined as the ratio between the aggregate’s central width (*W*_*c*_; corresponding to the minor axis) and major axis length (*L*_*t*_). With these metrics, by 24 hours postpairing, we observed highly consistent lengthwise compaction between aggregating spheroid pairs with normalized length (*L*_*t*_/*L*_0_) reaching 0.75 ± 0.02 (means ± SD), together with central width expansion reaching a width-length ratio (*W*_*c*_/*L*_*t*_) of 0.69 ± 0.03 (Fig. 2B). These findings indicate that freshly formed, 1-day-old hABSC spheroids, which do not yet present functional cilia on their exterior surface, could be used as CBBs capable of aggregating in a spatially controlled manner. On the basis of these findings, we theorized that these aggregates can then be matured into CiliaBots of desired geometries (AggreBots), in contrast with the spherical CiliaBots derived from one CBB (UniBots).

To generate more longitudinal observations of AggreBot morphology, we maintained rod-shaped AggreBots derived from two CBBs (denoted ${\text{CBB}}_{2}$ ) up until tissue maturation, imaging the aggregate at initial pairing (*t* + 0), 3 days after initial pairing (*t* + 3), and at day 20 postseeding as the AggreBots approached maturity. Furthermore, to evaluate for any alterations to the aggregation behavior resulting from the age of the CBB (number of days following hABSC seeding for CBB formation) at the time of initial pairing, we included in this test CBBs that were 1, 3, and 5 days old before being paired (the abbreviations M1, M3, and M5 are used to denote CBB age at the time point of first pairing and subsequent merging). We observed the same tendency toward lengthwise compaction and central width expansion with these experimental conditions as seen in the initial paired seeding experiments (Fig. 2C). However, the bulk of tissue reorganization occurs in the early days after pairing (i.e., normalized length in the M1 condition decreasing from 1.00 ± 0.02 to 0.68 ± 0.04 after the first 3 days of aggregation but decreasing further to only 0.56 ± 0.04 in the 16 days thereafter). Additionally, significant differences in both the normalized lengths (*P <* 0.001) and width-length ratios (*P <* 0.05) of the AggreBots stemming from CBB pairing at differing times were observed, with older CBB pairings tending to aggregate less in both length and width (Fig. 2C). Consistent with this, we observed a decrease in aggregation success rate corresponding to increased age of CBBs (table S1), indicating that pairing of CBBs at early time points is critical to the successful formation of AggreBots. We further observed similar values in the aggregation success rate across the tested donors (table S1).

To assess any effects of CBB aggregation on subsequent ciliogenesis on the exterior tissue surface, mature AggreBots were evaluated with cilia marker acetylated α-tubulin (Ac-α-Tub) (Fig. 2D). Ciliation of the AggreBots’ outer surface was then calculated from the middle slices of the AggreBots’ confocal *z*-stacks, measured as a percentage of the AggreBot surface covered in Ac-α-Tub signal (*27*, *33*). Staining of unaggregated CBBs that subsequently differentiated into UniBots was conducted as a control comparison. We found no significant difference in surface cilia coverage between the AggreBots of any prepairing CBB age when compared to UniBots (*P >* 0.05), indicating that the CBB aggregation process and resulting increase in tissue dimension do not interfere with multiciliated cell differentiation and apical cilia presentation (Fig. 2D). A further analysis of ciliary beat frequency (CBF) through kymograph analysis revealed comparable (*P* > 0.05) values in UniBots (7.69 ± 1.16 Hz) and in ${\text{CBB}}_{2}$ M1 AggreBots (7.14 ± 0.90 Hz), which were further in line with previous measurements of native bronchial cilia (fig. S2) (*34*).

Upon establishing the foundation of modular CBB aggregation with pairs delivering rod-shaped AggreBots, we pursued geometric configurations of increased complexity with ${\text{CBB}}_{3}$ triangle-shaped and ${\text{CBB}}_{4}$ diamond-shaped protocols, relying on the same well curvature as in the rod-shaped ${\text{CBB}}_{2}$ studies to bring the constituent CBBs into contact (Fig. 2E). Following the maturation period, triangle- and diamond-shaped AggreBots were achieved, reflective of the initial geometric configuration.

### Regulation of AggreBot motility by tissue geometric configuration

Following from our findings that CBB aggregation did not substantially affect surface cilia coverage, we theorized that a nonspherical AggreBot would exhibit increased motility compared to a spherical UniBot of equal cell count. The AggreBots, we reasoned, would have a larger surface area and thus an increased number of exterior-facing cilia for tissue propulsion while having near-equal tissue volume to the UniBot. We further hypothesized, circling back to our overarching objective, that the control over CiliaBot tissue geometry that aggregation provides would entail control over not only motility but also resulting locomotive patterns. To test for these hypotheses, we adopted the biomechanical assay of a 2D locomotion test, where mature CiliaBots would be allowed to move freely on a flat-bottom microplate (fig. S3) (*10*).

**Fig. 3.**
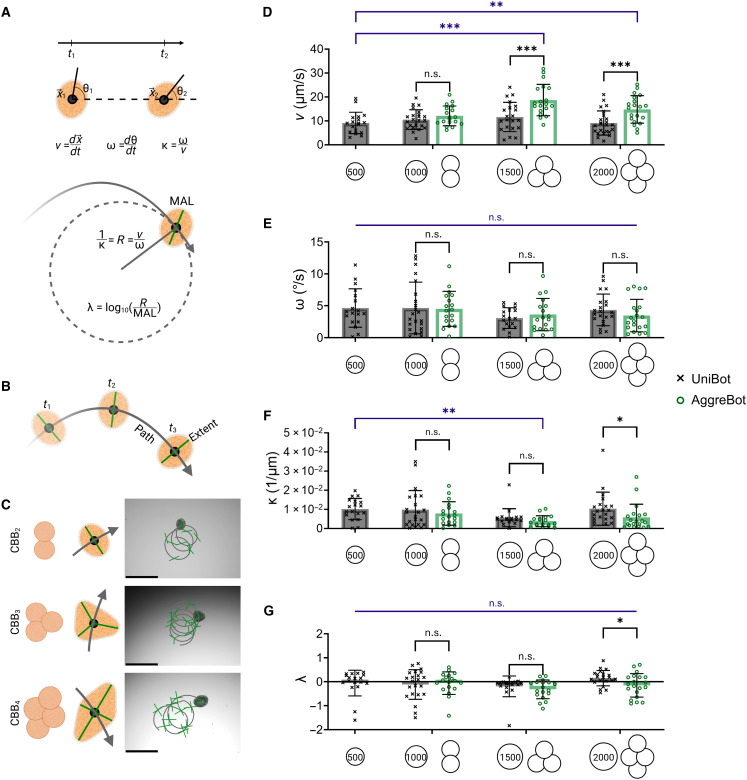
Characterization of AggreBot motility in comparison to UniBot motility. (**A**) Schematic depiction of quantification of translation velocity (*v*), rotational velocity (ω), path curvature (κ), and linear tendency (λ) through analysis of CiliaBot position (*x*), angle of orientation (θ), and major axis length (MAL). (**B**) Schematic depiction of the path-and-extent visualization method developed for displaying both CiliaBot position and angle of orientation over time. (**C**) Example path-and-extent depictions of ${\text{CBB}}_{2}$ , ${\text{CBB}}_{3}$ , and ${\text{CBB}}_{4}$ AggreBot motility, with associated schematics indicating the extent positioning for each AggreBot type. Scale bars, 500 μm. (**D** to **G**) Quantifications of CiliaBot translational velocity (*N* = 143) (D), rotational velocity (*N* = 135) (E), path curvature (*N* = 135) (F), and linear tendency (*N* = 135) (G) for ${\text{CBB}}_{2}$ , ${\text{CBB}}_{3}$ , and ${\text{CBB}}_{4}$ AggreBots in comparison with UniBots of the corresponding cell count. All data represent means ± SD. *P < 0.05, **P < 0.01, and ***P < 0.001. Two-way ANOVA with Tukey’s multiple comparisons test applied to the 1000-, 1500-, and 2000-cell CiliaBot groups (black) and one-way ANOVA with Tukey’s multiple comparisons test applied to the 500-cell UniBot group and all AggreBot groups (blue) in [(D) to (F)]. Created in BioRender. Ren, X. (2025) https://BioRender.com/ry5fhhz.

Before addressing these hypotheses, we sought to develop metrics for measuring CiliaBot motility. Most CiliaBots observed displayed a progressing spiral motion akin to a loop-de-loop (movies S2 to S4). These observations motivated the extraction of two primary motility metrics for CiliaBots: (i) the average translational speed, denoted *v*, representing the change in *XY*-position of the CiliaBot centroid over time; and (ii) the average rotational speed, denoted ω, representing the change in the orientation of the CiliaBot’s major axis over the same period (Fig. 3A). From these, the metric of average path curvature, denoted κ, was calculated, derived from the ratio of the two previous metrics (ω/*v*), representing the tightness of the spiral motion the CiliaBot generated and, by extension, a quantification of its motility pattern. We additionally created a metric of linear tendency, denoted λ, calculated as the common logarithm of the ratio between the radius of curvature (the inverse of κ) and the length of the CiliaBot’s major axis: An increase in this ratio is indicative of the CiliaBot tracing a wider, more linear-like arc. Concurrent with the development of these quantitative metrics, a visualization method of CiliaBot motility patterns was designed, known as the path-and-extent model, where the curve of the CiliaBot centroid is traced, denoting the “path” of locomotion, and the CiliaBot’s vertices, taken as a representation of the full space the CiliaBot traverses, represent the “extent” (Fig. 3B). Such a visualization method can be applied to both AggreBots and UniBots, delivering a distillation of any given motility pattern (Fig. 3C).

To test the hypothesis of increased surface area having a positive effect on motility, we stratified the overarching classifications of AggreBot (rod-shaped ${\text{CBB}}_{2}$ , triangle-shaped ${\text{CBB}}_{3}$ , and diamond-shaped ${\text{CBB}}_{4}$ ) and UniBot based on their hABSC counts at formation, ranging from 1000 to 2000 cells, from which any difference in motility between an AggreBot and a UniBot in a given cell “weight class” could only be reasonably attributed to tissue configuration and the resulting ciliated surface area. A significant increase in average translational speed in 1500-cell ${\text{CBB}}_{3}$ and 2000-cell ${\text{CBB}}_{4}$ AggreBots compared to their 1500- and 2000-cell UniBot counterparts was observed (*P <* 0.001) (Fig. 3D), while the speeds of 1000-cell ${\text{CBB}}_{2}$ AggreBots were not significantly different than their 1000-cell UniBot counterparts (*P* > 0.05). Across all tested cell counts, no significant differences were observed in average rotational speed (*P >* 0.05) (Fig. 3E). This observation extended to average path curvature and linear tendency, with the exception of the 2000-cell class (*P* < 0.05 and *P* < 0.05, respectively) (Fig. 3, F and G). These observations established that aggregation of CBBs into AggreBots can produce additional translational motility in excess of non-AggreBots of the same size.

To test the hypothesis of tissue geometry affecting the patterns of CiliaBot motility, a separate statistical test was conducted comparing the 500-cell UniBot (in essence, a ${\text{CBB}}_{1}$ AggreBot) and the three tested AggreBot forms to each other. When comparing the motility of the various AggreBot classes, the translational speed of the triangle-shaped ${\text{CBB}}_{3}$ (*P <* 0.001) and diamond-shaped ${\text{CBB}}_{4}$ (*P <* 0.01) AggreBots were found to be significantly elevated compared to the ${\text{CBB}}_{1}$ AggreBot (Fig. 3D). However, outside of a significantly decreased path curvature in the 1500-cell ${\text{CBB}}_{3}$ AggreBot compared to the ${\text{CBB}}_{1}$ AggreBot (*P <* 0.01) (Fig. 3F), minimal differences were observed in all other metrics outside of translational velocity (*P* > 0.05) (Fig. 3, E to G). These findings established that, while the manipulation of tissue geometry resulted in clear changes to CiliaBot motility, additional morphological levers would need to be investigated to achieve control over CiliaBot locomotion patterns.

### Controlling active cilia coverage leveraging immotile-cilia CBBs

**Fig. 4.**
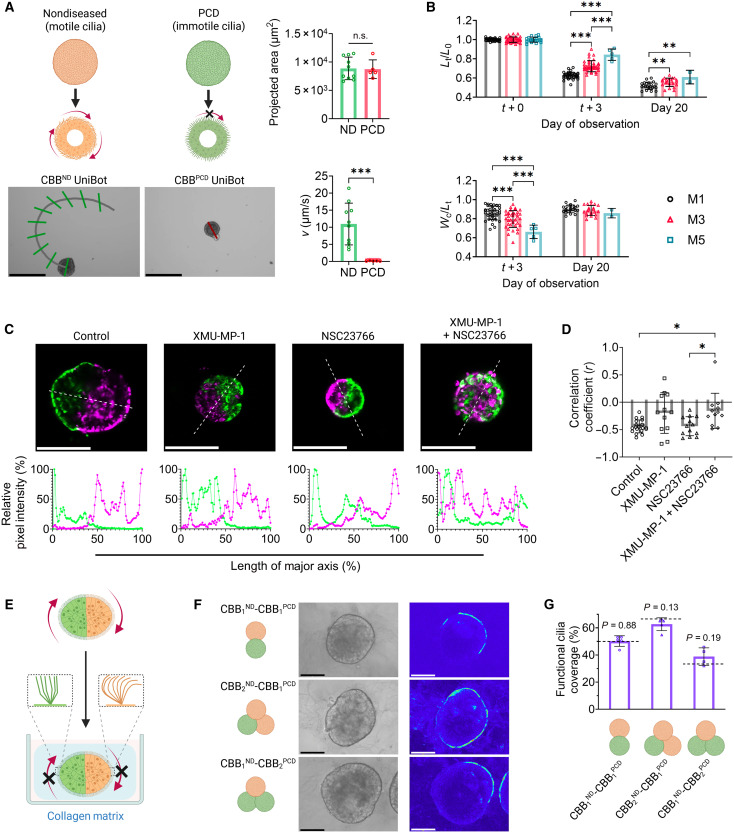
Incorporation of ${\text{CBB}}^{\text{PCD}}$ to generate hybrid AggreBots to control active cilia coverage. (**A**) Example path-and-extent depictions of ${\text{CBB}}^{\text{ND}}$ and ${\text{CBB}}^{\text{PCD}}$ UniBot motility, along with comparisons of morphology and motility between the UniBot classes (*N* = 15). Scale bars, 250 μm. (**B**) Quantification of normalized length (*L*_*t*_/*L*_*0*_) and width-length ratio (*W*_*c*_/*L*_*t*_) of hybrid ${\text{CBB}}_{1}^{\text{ND}}-{\text{CBB}}_{1}^{\text{PCD}}$ aggregation at CBB ages of 1, 3, and 5 days. Quantification was done immediately following CBB pairing (*N* = 94), 3 days following pairing (*N* = 80), and as the AggreBot approached tissue maturation at Day 20 (*N* = 46). (**C**) Example confocal slices of fluorescently dyed ${\text{CBB}}_{2}^{\text{ND}}$ AggreBot treated with 10 μM XMU-MP-1, 50 μM NSC23766, both, or neither, along with example quantifications of relative pixel intensity for green and red signal along the AggreBot’s major axis as determined by an image profile. Scale bars, 125 μm. (**D**) Quantification of correlation coefficients between paired green and red relative intensities across the AggreBot image profile when treated with XMU-MP-1, NSC23766, both chemicals, or neither (*N* = 56). (**E**) Schematic depiction of collagen embedding of AggreBots to arrest tissue-level motility and analyze functional cilia coverage in hybrid AggreBots. (**F**) Example depictions of collagen-embedded hybrid AggreBots, along with heatmaps of pixel intensity variation over the course of videotaping. Scale bars, 75 μm. (**G**) Quantification of functional cilia coverage in hybrid AggreBots, with associated *P* values for one-sample *t* tests comparing observed coverage to expected values (black dashed lines) (*N* = 14). All data represent means ± SD. *P < 0.05, **P < 0.01, and ***P < 0.001. Unpaired *t* test in (A), two-way ANOVA with Tukey’s multiple comparisons test in (B), one-way ANOVA with Tukey’s multiple comparisons test in (D), and one-sample t tests in (G). Created in BioRender. Ren, X. (2025) https://BioRender.com/a24t458.

With control over tissue geometry being established, we then investigated control over the surface coverage of active cilia as an additional “lever” with which to direct CiliaBot motility. In particular, we theorized that the ability to generate discrete “active” and “inactive” zones of cilia beating on the tissue surface would further augment motility control. To this end, we acquired hABSCs bearing genetic mutations in the *CCDC39* gene that cause PCD, a disorder that can affect both the structure and function of motile cilia; in the case of the *CCDC39* mutations, immotile cilia are produced (*28*–*32*, *35*, *36*). We observed that solitary CBBs formed from PCD hABSCs (denoted as ${\text{CBB}}^{\text{PCD}}$ ) underwent the same tissue reorganization and maturation seen in ND hABSC-derived CBBs (denoted as ${\text{CBB}}^{\text{ND}}$ ), ultimately producing “cilia-dead” UniBots that displayed limited motility when compared to “cilia-active” UniBots from ${\text{CBB}}^{\text{ND}}$ (Fig. 4A and movies S5 and S6), findings corroborated by our previous work (*27*).

To determine whether ${\text{CBB}}^{\text{PCD}}$ could be combined with ${\text{CBB}}^{\text{ND}}$ for hybrid AggreBot generation, we applied the same aggregation protocol previously used for ${\text{CBB}}_{2}^{\text{ND}}$ to the hybrid (denoted as ${\text{CBB}}_{1}^{\text{ND}}-{\text{CBB}}_{1}^{\text{PCD}}$ ). We observed similar aggregation dynamics in the hybrid to the ${\text{CBB}}_{2}^{\text{ND}}$ counterpart, including the bulk of lengthwise compaction and central width expansion taking place in the early days of aggregation. Later-aggregating CBB also produced more elongated and thinner AggreBots, like in the ${\text{CBB}}_{2}^{\text{ND}}$ experiment, although, by day 20, the differences in width-length ratio were not found to be statistically significant (*P >* 0.05) (Fig. 4B).

Upon demonstrating effective ${\text{CBB}}^{\text{PCD}}$-to-${\text{CBB}}^{\text{ND}}$ aggregation, we went on to assess whether CBBs merged within an AggreBot maintain distinct domains within the larger structure, which, if true, would be the key to achieving spatially controlled functional cilia coverage via guided placement of ${\text{CBB}}^{\text{PCD}}$ and ${\text{CBB}}^{\text{ND}}$ . To this end, we undertook investigations to assess both the structural and functional distinctiveness of CBB domains within AggreBots. Rod-shaped ${\text{CBB}}_{2}^{\text{ND}}$ AggreBots were generated, with the two ${\text{CBB}}^{\text{ND}}$ labeled with DiO (green) and DiD (red), respectively, before pairing. Intriguingly, despite being derived from the same hABSC source and culture conditions, the differentially labeled CBBs, while fusing into an AggreBot, did not intermix their constituent cells, instead maintaining a clear boundary between themselves (Fig. 4C).

Both cellular fluidity and tissue polarity are known to regulate cell migration in epithelial tissue development (*37*–*40*). To mechanistically investigate their involvement in boundary establishment between CBB domains within each AggreBot, we treated these dual-color CBB aggregates with XMU-MP-1, an activator of YES-associated protein (YAP) known to increase the fluidity of lung epithelial cells through the inhibition of a process known as “jamming,” and with NSC23766, an inhibitor of Rac1 guanosine triphosphatase, a protein responsible for the establishment of apical polarity in epithelial tissues (*37*–*40*). While neither small molecule alone disrupted the inter-CBB boundary significantly, their combination led to the intermixing of cells with distinct fluorescence labeling, as evidenced by the loss of negative correlation between regions of high green signal and high red signal in AggreBots receiving both XMU-MP-1 and NSC23766, suggesting boundary disruption (*P* < 0.05) (Fig. 4D).

With the structural distinctiveness of post-aggregation CBBs being established, we carried hybrid ${\text{CBB}}^{\text{ND}}$ - ${\text{CBB}}^{\text{PCD}}$ AggreBots to maturity to assess the functional distinctiveness of cilia activity. To this end, rod- and triangle-shaped hybrid AggreBots were generated and immobilized in collagen matrix to limit their 2D locomotion while allowing for close observation of surface cilia beating (Fig. 4E). In this environment, we observed distinct domains of cilia activity and inactivity, matching the configuration of ${\text{CBB}}^{\text{ND}}$ and ${\text{CBB}}^{\text{PCD}}$ as initially aggregated (Fig. 4F and movies S7 to S9). Quantitative analysis of the embedded AggreBots further corroborated the visual assessment (Fig. 4G), as the calculated functional cilia coverage for the ${\text{CBB}}_{1}^{\text{ND}}-{\text{CBB}}_{1}^{\text{PCD}}$ , ${\text{CBB}}_{2}^{\text{ND}}-{\text{CBB}}_{1}^{\text{PCD}}$ , and ${\text{CBB}}_{1}^{\text{ND}}-{\text{CBB}}_{2}^{\text{PCD}}$ configurations were not found to be significantly different from the theoretical coverage values predicted from the AggreBots’ compositions (50, 66.7, and 33.3%, respectively) (*P >* 0.05). This, together with the results of fluorescent CBB domain tracking (Fig. 4D), established the feasibility of using ${\text{CBB}}^{\text{PCD}}$ for spatial control of active cilia coverage on the AggreBot surface.

### Combinatorial control of tissue geometry and active cilia coverage to effect varied motility patterns

**Fig. 5.**
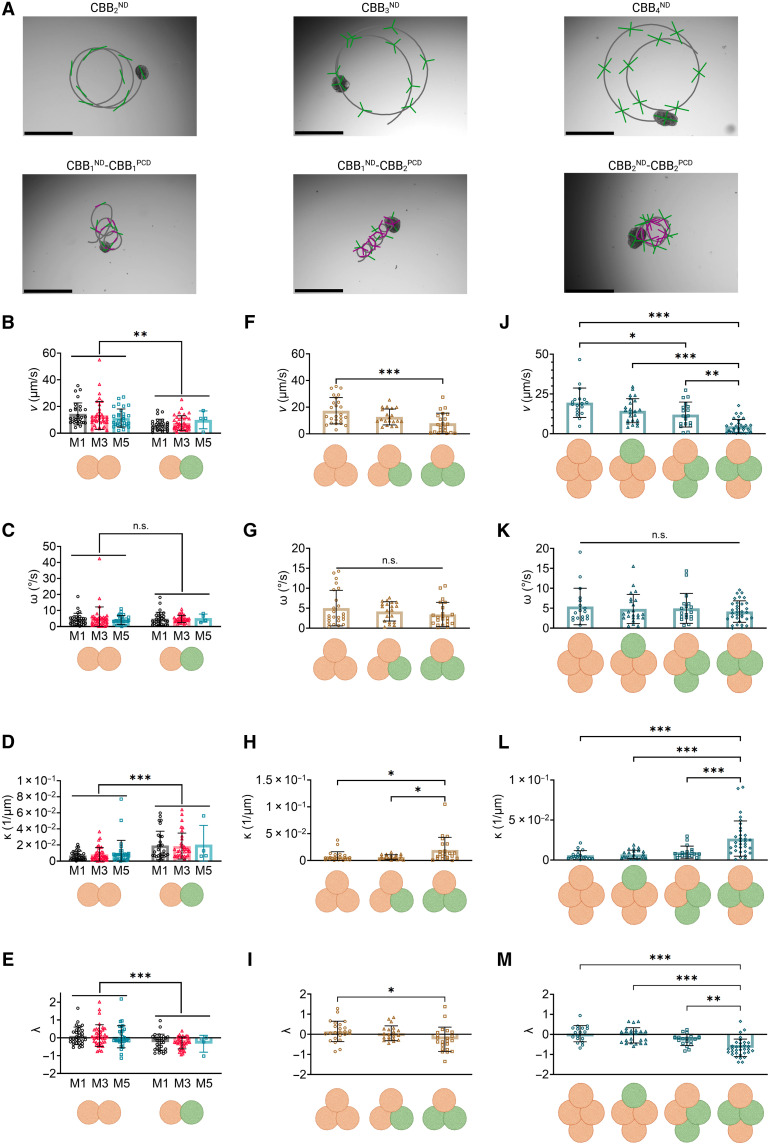
Characterization of hybrid AggreBot motility in comparison with all-${\text{CBB}}^{\text{ND}}$ AggreBots. (**A**) Example path-and-extent depictions of all-${\text{CBB}}^{\text{ND}}$ and hybrid ${\text{CBB}}_{2}$ , ${\text{CBB}}_{3}$ , and ${\text{CBB}}_{4}$ AggreBots. Scale bars, 500 μm. (**B** to **E**) Quantifications of ${\text{CBB}}_{2}$ AggreBot translational velocity (*N* = 168) (B), rotational velocity (*N* = 165) (C), path curvature (*N* = 165) (D), and linear tendency (*N* = 165) (E), comparing all-${\text{CBB}}^{\text{ND}}$ and hybrid AggreBots from 1-, 3-, and 5-day-old CBBs. Orange spheroids represent ${\text{CBB}}^{\text{ND}}$ , while green spheroids represent ${\text{CBB}}^{\text{PCD}}$ . (**F** to **I**) Quantifications of ${\text{CBB}}_{3}$ AggreBot translational velocity (*N* = 67) (F), rotational velocity (*N* = 66) (G), path curvature (*N* = 66) (H), and linear tendency (*N* = 66) (I), comparing all-${\text{CBB}}^{\text{ND}}$ and hybrid ( ${\text{CBB}}_{2}^{\text{ND}}-{\text{CBB}}_{1}^{\text{PCD}}$ and ${\text{CBB}}_{1}^{\text{ND}}-{\text{CBB}}_{2}^{\text{PCD}}$ ) AggreBots. Orange spheroids represent ${\text{CBB}}^{\text{ND}}$ while green spheroids represent ${\text{CBB}}^{\text{PCD}}$ . (**J** to **M**) Quantifications of ${\text{CBB}}_{4}$ AggreBot translational velocity (*N* = 85) (J), rotational velocity (*N* = 85) (K), path curvature (*N* = 85) (L), and linear tendency (*N* = 85) (M), comparing all-${\text{CBB}}^{\text{ND}}$ and hybrid ( ${\text{CBB}}_{3}^{\text{ND}}-{\text{CBB}}_{1}^{\text{PCD}}$ , chiral ${\text{CBB}}_{2}^{\text{ND}}-{\text{CBB}}_{2}^{\text{PCD}}$ , and symmetric ${\text{CBB}}_{2}^{\text{ND}}-{\text{CBB}}_{2}^{\text{PCD}}$ ) AggreBots. Orange spheroids represent ${\text{CBB}}^{\text{ND}}$ , while green spheroids represent ${\text{CBB}}^{\text{PCD}}$ . All data represent means ± SD. *P < 0.05, **P < 0.01, and ***P < 0.001. Two-way ANOVA with Tukey’s multiple comparisons test in [(B) to (E)] and one-way ANOVA with Tukey’s multiple comparisons test in [(F) to (M)]. Created in BioRender. Ren, X. (2025) https://BioRender.com/xd7wxlh.

With control over both tissue geometry and active cilia coverage established, we sought to quantify the effects of combinatorial manipulation of these independent variables on AggreBot motility patterns. To this end, we constructed rod-, triangle-, and diamond-shaped AggreBots with either all ${\text{CBB}}^{\text{ND}}$ or a combination of ${\text{CBB}}^{\text{ND}}$ and ${\text{CBB}}^{\text{PCD}}$ and compared their 2D locomotion. To assess and visualize motility, the path-and-extent visualization method was modified to allow a clear distinction of AggreBot types via green versus magenta centroid-vertex segments, corresponding to researcher input, indicating ${\text{CBB}}^{\text{ND}}$ versus ${\text{CBB}}^{\text{PCD}}$ domains within the AggreBot, respectively (Fig. 5A and movies S10 to S15).

Starting with the rod-shaped AggreBots, we observed a significant decrease in average translational speed in the ${\text{CBB}}_{1}^{\text{ND}}-{\text{CBB}}_{1}^{\text{PCD}}$ configuration compared to the ${\text{CBB}}_{2}^{\text{ND}}$ (*P <* 0.01), which proved indicative of a trend of ${\text{CBB}}^{\text{PCD}}$ introduction, leading to a decrease in translational speed [and an accompanying increase in average path curvature (*P* < 0.001) and decrease in linear tendency (*P* < 0.001)]. This trend is also seen in triangle-shaped AggreBots, where ${\text{CBB}}_{1}^{\text{ND}}-{\text{CBB}}_{2}^{\text{PCD}}$ showcase decreased translational speed (*P <* 0.001), increased path curvature (*P <* 0.05), and decreased linear tendency (*P* < 0.05) when compared to ${\text{CBB}}_{3}^{\text{ND}}$ AggreBots, with ${\text{CBB}}_{2}^{\text{ND}}-{\text{CBB}}_{1}^{\text{PCD}}$ AggreBots exhibiting motility patterns between these extremes (Fig. 5, F, H, and I). Of particular note are the differing motility results within the tested diamond-shaped AggreBots. While the same trend seen in rod- and triangle-shaped AggreBots was present, the increased number of constituent CBBs per diamond-shaped AggreBot allows for a higher number of permutations in which the ${\text{CBB}}^{\text{ND}}$ and ${\text{CBB}}^{\text{PCD}}$ could be arranged, leading to two separate, tested diamond AggreBot configurations with an overall composition of ${\text{CBB}}_{2}^{\text{ND}}-{\text{CBB}}_{2}^{\text{PCD}}$ (Fig. 5, J to M). Here, we found that the different permutations (in particular, the locations of the ${\text{CBB}}^{\text{ND}}$ within the larger structure) resulted in significantly altered translational speed (*P <* 0.01), path curvature (*P <* 0.001), and linear tendency (*P* < 0.01). The chiral ${\text{CBB}}_{2}^{\text{ND}}-{\text{CBB}}_{2}^{\text{PCD}}$ variant was more likely to adopt a loop-de-loop locomotion pattern (Fig. 5A and movie S15). In contrast, the symmetric variant instead displayed minimal translational speeds but substantial rotational speeds, indicating rotation in place (movie S16).

**Fig. 6.**
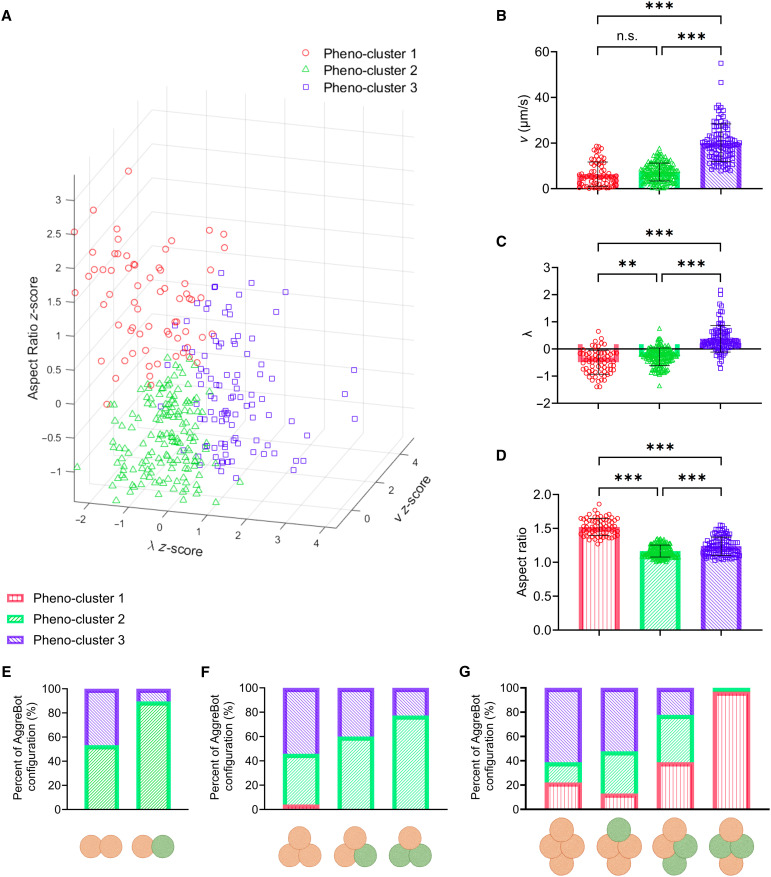
Phenotypic clustering of AggreBot motility and behavior. (**A**) 3D scatter plot of AggreBot translational velocity, linear tendency, and aspect ratio, with each variable normalized and reported as a *z*-score (*N* = 323). Unsupervised, fuzzy *c*-means clustering was used to separate the plot into pheno-clusters 1 to 3 (*n* = 67, *n* = 154, and *n* = 102, respectively). (**B** to **D**) Quantifications of translational velocity (*N* = 323) (B), linear tendency (*N* = 323) (C), and aspect ratio (*N* = 323) (D), comparing AggreBots of each pheno-cluster. (**E** to **G**) Quantifications of the proportions of each AggreBot type that belong to a given pheno-cluster in ${\text{CBB}}_{2}$ (*N =* 59) (E), ${\text{CBB}}_{3}$ (*N* = 66) (F), and ${\text{CBB}}_{4}$ (*N* = 85) AggreBots (G). Pheno-cluster color-coding (red, green, and blue for pheno-clusters 1, 2, and 3, respectively) is consistent across all panels. Proportion quantifications in (E) to (G) represent AggreBots from 1-day-old CBBs. All data in [(B) to (D)] represent means ± SD. **P < 0.01, and ***P < 0.001. One-way ANOVA with Tukey’s multiple comparisons test in [(B) to (D)]. Created in BioRender. Ren, X. (2025) https://BioRender.com/h67vjf0.

To delineate the behavioral patterns of the designed AggreBots and further investigate the potential mechanisms that dictate the locomotive trends that we observed above with the introduction of ${\text{CBB}}^{\text{PCD}}$ , we undertook unsupervised clustering of the filmed AggreBots based on phenotypic outcomes, specifically the motility metrics of translational velocity and linear tendency, combined with the aspect ratio of the construct, defined as the ratio between the major and minor axes lengths, to ascertain any clustering present around CBB count. The aggregate dataset was then normalized and partitioned using fuzzy *c*-means clustering, which identified three phenotypic clusters (pheno-clusters) within the data (Fig. 6A). Pheno-cluster 1 was observed to contain AggreBots of significantly greater aspect ratio than either pheno-cluster 2 or 3 (*P* < 0.001) while also exhibiting significantly decreased translational velocity from pheno-cluster 3 (*P* < 0.001) and decreased linear tendency from pheno-cluster 2 or 3 (*P* < 0.01 and *P* < 0.001, respectively) (Fig. 6, B to D). Additionally, pheno-cluster 2 AggreBots were observed to have the smallest aspect ratios (*P* < 0.001). In contrast, pheno-cluster 3 AggreBots had a significantly higher translational velocity and linear tendency than either pheno-cluster 1 or 2 (*P* < 0.001) (Fig. 6B to D).

With these three phenotypic classes broadly defined, we then identified the proportions of each AggreBot design that were of a given pheno-cluster. The largest proportions of AggreBots in pheno-cluster 1 were found in the hybrid ${\text{CBB}}_{4}$ configurations, particularly both the chiral and symmetric ${\text{CBB}}_{2}^{\text{ND}}-{\text{CBB}}_{2}^{\text{PCD}}$ variants (38.9 and 97.0% being pheno-cluster 1, respectively) (Fig. 6E). Pheno-cluster 2 was the dominant behavioral pattern for the ${\text{CBB}}_{1}^{\text{ND}}-{\text{CBB}}_{1}^{\text{PCD}}$ , ${\text{CBB}}_{2}^{\text{ND}}-{\text{CBB}}_{1}^{\text{PCD}}$ , and ${\text{CBB}}_{1}^{\text{ND}}-{\text{CBB}}_{2}^{\text{PCD}}$ AggreBots (89.7, 60.0, and 77.3%, respectively) (Fig. 6, E and F). Last, for every given CBB count, pheno-cluster 3 was most prominent in the all-${\text{CBB}}^{\text{ND}}$ configurations, with that proportion incrementally decreasing with the introduction of ${\text{CBB}}^{\text{PCD}}$ (Fig. 6, E to G).

## DISCUSSION

In this work, we established the AggreBot as a cutting-edge platform for augmenting control over CiliaBot motility phenotypes through direct modulation of tissue geometry and active cilia coverage. To accomplish this, we established a modular approach to spatially control the aggregation of CBBs that serve as building blocks for higher-order CiliaBots; this was accomplished while maintaining homogeneous coverage of motile cilia on the CiliaBot’s exterior surface upon tissue maturation. We further established control over the extent of functional cilia coverage on tissue surface, incorporating PCD-derived, cilia-dead CBBs into the aggregation protocol, to deliver controllable cilia activity distribution and further pronounced phenotypic alterations to motility patterns.

The research herein provides a comprehensive understanding of the aggregation behavior of epithelial spheroids derived from human airway stem cells, a phenomenon that was previously observed (*10*). In making this process the focal point of our work, we have found spheroid aggregation to be highly reproducible and characterizable, allowing us to additionally understand the time-dependent nature of this aggregation process. The discovery that the age of CBBs can modulate both the success rate and the extent of aggregation before aggregation indicates to us the possibility that the cellular composition of CBBs influences epithelial aggregation behavior as they progressively differentiate from an initial stem cell state. The CBB aggregation platform and associated morphological characterization pipeline described here, together with our findings, could serve as the basis for future research to further expand our abilities to control the manner and extent of CBB aggregation and, ultimately, AggreBot geometry.

The long-term maintenance of boundaries between constituent CBB domains post-aggregation is another fascinating advancement in our understanding of epithelial merging. Activation of the YAP-Taz signaling pathway has been shown to encourage epithelial cell fluidity, while inhibition of Rac1 hampers the establishment of apical-basolateral polarity (*37*–*40*). The finding that the combination of these effectors ultimately disrupted the inter-CBB boundary (with either chemical alone proving insufficient) suggests multiple inherent cellular mechanisms function in a redundant manner to prevent epithelial migration across predefined tissue domains, which further speaks to the robust nature of the AggreBot platform for spatial patterning of distinct tissue domains that can be maintained throughout maturation.

Furthermore, this work pioneered controlling CiliaBot geometry through the modular assembly of uniform CBBs. The regularity of the underlying CBBs, in terms of cellular composition, tissue dimension, differentiation potential, and aggregation behavior, is crucial to the AggreBot platform introduced in this research, without which shape planning and resulting aggregation protocols would not have been possible. Both the current and prior investigations have demonstrated that overnight culture of hABSCs under cell-repellent conditions in the absence of extracellular matrix support is a simple yet effective way to generate CBBs with these desired features (*1*, *10*, *27*). Additionally, we demonstrate effective CBB aggregation without sacrificing uniform cilia coverage on the mature tissue’s outer surface (*27*). Together, these pillars allow for the delivery of high-level shape control in mature AggreBots, resulting in enhanced translational motility over UniBots with the same cell count. This increased power-to-weight ratio is especially promising when considering the scalability of CiliaBot motility. To deliver CiliaBots of greater size and higher constituent CBB number in the future, additional manufacturing capacities, such as aspiration-assisted bioprinting to spatially pattern tissue spheroids, may be required to augment the throughput and control of AggreBot generation (*41*–*44*). A further consideration is that as AggreBot complexity and capability increase, future research in this discipline may be confronted with ethical concerns, potentially regarding whether such constructs constitute living entities. Such challenges could be further compounded due to the use of human cells for tissue construction and will need to be carefully addressed to ensure ethical compliance (*1*, *45*).

Prior reports have shown that CiliaBots can be generated with spontaneous variations in morphology (i.e., spherical versus elongated) and surface cilia (i.e., fully covered versus polarized), which can, in turn, be correlated with specific motility patterns (*10*). These findings propelled us not only to observe active cilia coverage but also to directly control it through the use of ${\text{CBB}}^{\text{PCD}}$ bearing genetic mutations resulting in nonfunctional cilia to designate cilia-dead domains. Through the geometrically controlled aggregation of ${\text{CBB}}^{\text{PCD}}$ with the cilia-active ${\text{CBB}}^{\text{ND}}$ , we demonstrated hybrid AggreBots with robust control over active cilia coverage. This configurable type of CiliaBot was made possible through the combination of the following foundational observations. First, CBBs made from ND and PCD cells behaved similarly in terms of CBB formation, differentiation, and aggregation, resulting in tissue domains that are structurally near indistinguishable but deliver substantially different functional outputs, a finding corroborated by previous research (*27*). Second, CBBs showcased an inherent ability to maintain an internal boundary post-aggregation. These unique features allow mature AggreBots of both homogeneous and hybrid compositions to faithfully represent their initial designs and predictably diversify CiliaBot motility patterns. Our efforts in AggreBot development were further supported by our established analytical pipeline to capture CiliaBot motility as a combination of translation and rotation. We anticipate that this visual distillation of AggreBot behavior will allow for the rapid identification of desirable CBB compositional groupings in the future. Additionally, we have observed the aggregation behavior to be applicable across sourced donors, given the aggregation success rates seen in ${\text{CBB}}^{\text{ND}}\;$AggreBots (across healthy donor sources) and those of the hybrid variety.

One of the most captivating findings of this study is that CiliaBot motility and behavior can be varied through the permutation of CBB positions within the AggreBot, as evidenced by the differences in the motilities of symmetric and chiral ${\text{CBB}}_{2}^{\text{ND}}-{\text{CBB}}_{2}^{\text{PCD}}$ AggreBots. This observation further underscores the versatility of the AggreBot platform when designing higher-order CiliaBots. Simply by varying the order in which the CBBs were aggregated, additional locomotive behaviors were identified and replicated. This finding highlights a third lever of structural control in CiliaBot generation that directly emerges out of control over geometry and cellular composition: tissue permutation. With modulation of this third lever, we achieved control of active cilia distribution along with overall coverage. Through these levers provided by the AggreBot platform, we open the door to constructing living propeller systems with preplanned, patterned ciliation, rather than the ubiquitous ciliation of UniBots, serving as a key step forward in controlling CiliaBot motility patterns.

The CiliaBots generated throughout this research present a broad range of locomotion characteristics, particularly with regard to the “linearity” of their traversed path: Certain AggreBots displayed linear-like motility within the timescale observed (movies S17 to S19), while others showcased highly circular motion (movie S16). In controlling CBB type, we uncovered a general negative correlation between active cilia coverage and AggreBot path linearity, along with manipulation of the radius of curvature over more than three orders of magnitude. Additionally, through the use of unsupervised clustering, we uncovered potential synergistic effects on motility patterns that arise from tissue composition, size, and geometry, which suggest a combinatorial mechanism behind CiliaBot behavior.

At this current time, the AggreBot platform has generated strong correlations between controllable aspects of AggreBot morphology and phenotypic alterations to AggreBot locomotion, allowing us to reduce the heterogeneity of CiliaBot motility when compared to the conventional UniBot. However, while dominant single patterns of motility have been identified in certain AggreBot configurations, a given AggreBot type is still liable to generate multiple locomotion phenotypes, indicating a lower signal-to-noise ratio than would be ideal. Challenges for the platform to overcome in the near term would be focused on improving the translation fidelity between the designer morphological control displayed in this study and end-result motility to truly predict phenotypic outcomes, along with pursuing AggreBot configurations that can controllably achieve additional output behaviors, particularly motility patterns that can consistently increase linear tendency. The solution to improving the applicability of the AggreBot platform may, therefore, lie in increasing the size and complexity of the constructed tissue itself. In moving beyond a maximum of four CBBs, exponentially increasing the available AggreBot permutations, future work should aim to expand upon the observed trends and discretize the observed pheno-clusters and their spectra of linearity into more predictable domains depending on AggreBot configuration, leveraging the modular, scalable design platform that the AggreBot provides to move past the progressing spiral motion paradigm observed in this research and previous works (*10*, *27*).

Prior work has shown great potential for CiliaBots to serve as cell-based, deployable therapeutics, with a capability for traversing complex geometries and zeroing in on damaged tissue (*10*). To bring this vision closer to reality, the AggreBot platform will allow for the critically needed diversification and optimization of CiliaBot design, with the ultimate potential to achieve desired, user-controlled motility. Beyond investigating CiliaBot behavior in an aqueous environment as described in this and prior works (*10*, *27*), it would also be of great interest to understand how they interact with mucus-like, viscoelastic environments; this regime is likely to be more challenging but more clinically relevant for several diseases (PCD, cystic fibrosis, chronic obstructive pulmonary disease, etc.). This also ties into the prospective use of patient-derived cells and genetic engineering of the AggreBot’s constituent cells to express therapeutic reagents of interest, such as recombinant proteins. Toward these future goals, the tools, techniques, and findings of this work substantially advance the capabilities and reproducibility of CiliaBots, enabling future studies to move beyond the bespoke design of biobots for individualized purposes toward true engineered design of robust, motile CiliaBots.

## MATERIALS AND METHODS

### Culture of hABSCs

hABSCs from ND donors were obtained from Lonza (CC-2540) and Promocell (C-12640). hABSCs carrying PCD-associated genetic mutations in the *CCDC39* gene were isolated from de-identified tissues with permission from the institutional review board at Washington University in Saint Louis (Institutional Review Board, no. 201705095) and obtained from participants who provided informed written consent. Age/sex information for each cell donor can be found in table S2. Both ND- and PCD-hABSCs [postnatal day 3 (P3) to P5)] were maintained at 37°C with 5% CO_2_ in bronchial epithelial cell growth medium (Lonza, CC-3171) supplemented with 1 μM A83-01 (Sigma-Aldrich, SML0788), 5 μM Y-27632 (Cayman Chemical, 129830-38-2), 0.2 μM DMH-1 (Tocris, 4126), and 0.5 μM CHIR99021 (REPROCELL, 04000402) on T-25 flasks (Greiner Bio-One, 690175) precoated with conditioned medium from 804G cells, with initial plating density of 3500 cells/cm^2^ (*27*, *46*). A full-medium change was conducted every 2 days after plating.

### Generation of CBBs and differentiation into UniBots

hABSCs (P3 to P5) between 60 and 75% confluency were lifted from T-25 flasks with TrypLE (Thermo Fisher Scientific, 12563011) and resuspended in differentiation medium composed of PneumaCult-ALI Medium (STEMCELL Technologies, 07925), which is further supplemented with 1 μM A83-01 and 5 μM Y-27632 to improve overall cell viability (*47*). Single-cell suspensions of hABSCs were then seeded into U-bottom, 96-well, cell-repellent microplates (Greiner Bio-One, 655970) at the desired number of cells (500, 1000, 1500, or 2000) per well and allowed to cluster overnight. The resulting hABSC spheroid was referred to as a CBB. To establish a naming convention for these AggreBots to clearly indicate their composition, we used superscripts to denote cell type and subscripts to denote the number of CBBs within an AggreBot. Specifically, CBBs derived from ND- and PCD-hABSCs were referred to as ${\text{CBB}}^{\text{ND}}$ and ${\text{CBB}}^{\text{PCD}}$ , respectively. ${\text{CBB}}_{2}$ and ${\text{CBB}}_{4}$ , for example, would indicate AggreBots formed from two or four constituent CBBs, respectively. To produce a spherical CiliaBot consisting of a single CBB (referred to as UniBot), each solitary CBB was further differentiated for 3 to 4 weeks with half-medium changes every other day and incubated at 37°C with 5% CO_2_ to reach full maturity (*27*).

### Aggregation of CBB and differentiation into AggreBots

To engineer AggreBots, a defined number of 1-day-old CBBs were placed in the same well within a U-bottom, 96-well, cell-repellent microplate in a geometrically controlled manner and allowed to aggregate and further differentiate for 3 to 4 weeks to reach full maturity under the conditions described above for UniBots. Rod- and triangle-shaped AggreBots were generated through controlled aggregation of two and three CBBs, respectively. For diamond-shaped AggreBots, to enable consistent placement of CBBs during aggregation, two 1-day-old CBBs were allowed to aggregate first overnight, followed by the introduction of two additional, 2-day-old CBBs. AggreBots were produced either all from ${\text{CBB}}^{\text{ND}}$ or from a combination of ${\text{CBB}}^{\text{ND}}$ and ${\text{CBB}}^{\text{PCD}}$ as specified in Results. To investigate how CBB age influences aggregation behavior, rod-shaped AggreBots were also aggregated from two CBBs at 3 and 5 days of age.

### Morphological analysis of rod-shaped AggreBots

Rod-shaped AggreBots derived from two CBBs were imaged under bright field using an EVOS M7000 microscope (Thermo Fisher Scientific) immediately after CBB pairing, 3 days after aggregation, and as the AggreBots approached maturity (20 days after initial CBB formation). The 2D projections of the AggreBots from these images were manually traced and analyzed using custom MATLAB programs that extracted the major axis of the projection (representing the AggreBot length) and the bisecting line of the projection’s area running perpendicular to the major axis (representing the AggreBot width) at each of the time points of imaging, which were further used to calculate the normalized length and width-length ratio. Data for a given aggregation time point were collected from two to three separate experiments.

### CiliaBot motility analysis and visualization

For analysis and visualization, mature AggreBots and UniBots (collectively CiliaBots) were each transferred to a separate well within flat-bottom, 96-well microplates with culture medium supplemented with 25 mM Hepes (Gibco, 15630080) to enable pH control outside of the incubator environment. Videotaping of CiliaBot motility was performed at room temperature, with videos captured between 5 and 30 frames per second (FPS) using a Nikon TS2 microscope equipped with a Moment complementary metal-oxide semiconductor camera.

CiliaBot videos were analyzed using custom MATLAB programs that automatically extracted the 2D projections of the CiliaBots across the entire runtime via edge detection to identify areas in the video frames of high pixel contrast as a result of CiliaBots being noticeably darker than the background, before filling in the space between detected edges to generate a binary mask representing the CiliaBot. From these projections, the *XY*-position of the centroid and the major axis orientation were determined through blob analysis of the binary mask. These metrics were further used to calculate the average translational velocity, average rotational velocity, average path curvature, and average linear tendency (Fig. 2A). A Savitzky-Golay smoothing filter was applied to the outputs of major axis orientation to reduce the influence of noise on rotational velocity calculations. Data for a given CiliaBot configuration (CBB count, type, size, etc.) were collected from two to three separate experiments.

To further reveal the dynamic relationship between CiliaBot rotation and position, the path-and-extent visualization method was developed to transduce quantitative motility data into a single, concise image. To enable this visualization, the *XY*-positions of the CiliaBot vertices were further obtained through blob analysis of the CiliaBots’ binary mask: for UniBots and rod-shaped AggreBots, the vertices of the major axis; for triangle-shaped AggreBots, the three centroid-vertex line segments; and for diamond-shaped AggreBots, the vertices of both the major and minor axes. These additional obtained values were used to depict the path-and-extent of a given CiliaBot’s motility pattern, superimposed on a freeze-frame of the video’s end. The path of the visualization stems from plotting the *XY*-position of the centroid over time. The extent stems from depicting the major axis, minor axis, and/or centroid-vertex line segments every 30 s of the video’s runtime. When used for hybrid AggreBots, regions of immotile cilia were visually identified during videotaping, with the identified vertex being highlighted with magenta extent to contrast the green extent denoting regions of motile cilia.

### Immunofluorescence staining and imaging

CiliaBots were fixed with 4% paraformaldehyde for 1 hour at 4°C and washed with phosphate-buffered saline (PBS) with 0.1% Tween 20 (PBST). For immunofluorescence staining, CiliaBots were permeabilized with 1% Triton X-100 in PBS for 45 min, blocked with 1% bovine serum albumin in PBS, and incubated with the mouse anti–Ac-α-Tub (Sigma-Aldrich, T6793) overnight at 4°C. Following a PBST wash, they were then incubated with donkey anti-mouse immunoglobulin G (H+L) secondary antibody, Alexa Fluor 647 (Thermo Fisher Scientific, A-31571), for 45 min at room temperature, and nuclear counterstained with 4′,6-diamidino-2-phenylindole (DAPI) (Thermo Fisher Scientific, D1306). *Z*-stack images of stained CiliaBots were captured on a Nikon A1 confocal microscope (*27*).

### Morphological cilia coverage analysis

From *z*-stack images of UniBots and ${\text{CBB}}_{2}$ AggreBots (M1, M3, and M5) stained with Ac-α-Tub and DAPI, three *z*-slices in or near the CiliaBot’s midsection were selected. Pixels of DAPI expression were used to generate a convex hull representing the tissue area, from which the centroid was calculated. From the calculated centroid, each *z*-slice was divided into 1° angular segments with an axis of rotation perpendicular to the image slice (Fig. 1D). Last, using a custom MATLAB program, the presence of Ac-α-Tub signal outside of the DAPI convex hull, limiting search only to signal found on the CiliaBot’s exterior surface, was evaluated in each angular segment, with a running tally being kept of which segments of Ac-α-Tub signal were above a threshold set to 10% of the maximum Ac-α-Tub signal intensity in the image. The count of angular segments containing Ac-α-Tub signal was then divided by 360 to calculate the morphological cilia coverage (*27*, *33*). Data for UniBots and AggreBots were collected from two separate experiments.

### Collagen embedding of CiliaBots for functional cilia coverage analysis and CBF calculation

AggreBots were transferred to a 1.5-ml Eppendorf tube and kept on ice. Ice-cold collagen type 1 (Advanced BioMatrix, 5225) was neutralized and added to the Eppendorf tube at a concentration of 2 mg/ml, with 200 μl of this mixture of AggreBot and pre-gel collagen being transferred to a glass-bottom dish (Mattek, P35G-1.5-14-C). This dish was kept on ice for 10 min to allow the AggreBots to settle at the bottom. The Mattek dish was then incubated at 37°C for 10 min to allow collagen gelation before adding 1 ml of differentiation medium. Video recording of cilia beating was captured using an EVOS M7000 microscope with a 40× objective camera at 30 FPS.

For functional cilia coverage analysis, a batch of videos of collagen-embedded ${\text{CBB}}_{1}^{\text{ND}}-{\text{CBB}}_{1}^{\text{PCD}}$ , ${\text{CBB}}_{2}^{\text{ND}}-{\text{CBB}}_{1}^{\text{PCD}}$ , and ${\text{CBB}}_{1}^{\text{ND}}-{\text{CBB}}_{2}^{\text{PCD}}$ AggreBots was first processed with a video stabilization tool (www.onlineconverter.com/stabilize-video) to eliminate motion within the video stemming from jitter. A Gaussian filter of 0.5 deviations was further applied to smooth any remaining artifacts. Following this preprocessing, the range of pixel intensities throughout the captured video was calculated from each pixel and converted into a heatmap to identify regions of high motion activity, from which a custom MATLAB program identified the presence and size of the active cilia domain through the identification of high-intensity pixels in 360 angular segments along the surface of the AggreBots.

For the CBF calculation, a batch of videos of collagen-embedded UniBots and ${\text{CBB}}_{2}$ M1 AggreBots were resliced in ImageJ along a line drawn parallel to the CiliaBot surface to obtain a space-time encoding of pixel intensity, after which an image profile along the time axis was obtained and plotted, providing the kymograph. The number of peaks in this kymograph was then counted and divided by the length of the video to obtain the calculated CBF in Hz. For multi-CBB AggreBots, the calculated CBF values of each CBB were averaged to obtain an overall CBF. Data for UniBots and AggreBots were collected from two separate experiments.

### AggreBot boundary integrity analysis

To assess boundary integrity, AggreBots were formed from two CBBs stained with DiD (Thermo Fisher Scientific, V22887) and DiO (Biotium, 60015) fluorescent dyes at 1 μM, respectively. These stained CBBs were aggregated on day 1 and treated with either YAP activator XMU-MP-1 (Tocris Bioscience, 6482) at 10 μM, Rac1 inhibitor NSC23766 (MilliporeSigma, SML0952) at 50 μM, or both. Untreated CBBs were aggregated as a control group. Two weeks following aggregation, *z*-stack images of the resulting immature AggreBots were captured on a Zeiss LSM 700 confocal microscope, from which the middle image slice was selected. An image profile running the AggreBot’s major axis length was then extracted using a custom MATLAB program, and the pixel intensity relative to the maximum of both green and red channels was charted. The correlation coefficient between the green and red pixel intensity pairs across the major axis length was then calculated. Data for each treatment condition were collected from two to three separate experiments.

### AggreBot phenotypic clustering

Translational velocity, linear tendency, and aspect ratio data were collected for all AggreBots that appear in Fig. 5 (E, I, and M). The values in these three metrics were normalized to obtain a *z*-score for each. The *z*-scores were then plotted into 3D space, and fuzzy *c*-mean clustering was undertaken using the Fuzzy Logic Toolbox in MATLAB. The output fuzzy partition matrix was used to split the data into three clusters on the basis of the maximum degree of membership for a given phenotypic cluster (pheno-cluster).

### Quantification and statistical analysis

All data were displayed as means ± SD. Statistical tests used throughout this study were two-way analysis of variance (ANOVA) with Tukey’s multiple comparisons test, one-way ANOVA with Tukey’s multiple comparisons test, unpaired *t* test, and one-sample *t* test. All analyses were performed using GraphPad Prism. *P < 0.05, **P < 0.01, and ***P < 0.001. *n* values for all experiments can be found in table S3 for improved clarity, while population-wide *N* values can be found in the figure descriptions.
