# Supplementary material for: AggreBots: Configuring CiliaBots through guided, modular tissue aggregation
Source: Sci Adv. 2025 Sep 26;11(39):eadx4176. doi: 10.1126/sciadv.adx4176 (PMC12467051; doi:10.1126/sciadv.adx4176)
Supplement: Supplementary file 1 — Figs. S1 to S3 Tables S1 to S3 Legends for movies S1 to S19 [file sciadv.adx4176_sm.pdf]

Science Advances
AAAS

# Supplementary Materials for

## AggreBots: Configuring CiliaBots through guided, modular tissue aggregation

Dhruv Bhattaram *et al.*

Corresponding author: Xi Ren, xiren@cmu.edu

*Sci. Adv.* **11**, eadx4176 (2025)
DOI: 10.1126/sciadv.adx4176

**The PDF file includes:**

Figs. S1 to S3
Tables S1 to S3
Legends for movies S1 to S19

**Other Supplementary Material for this manuscript includes the following:**

Movies S1 to S19

## Supplementary Figures

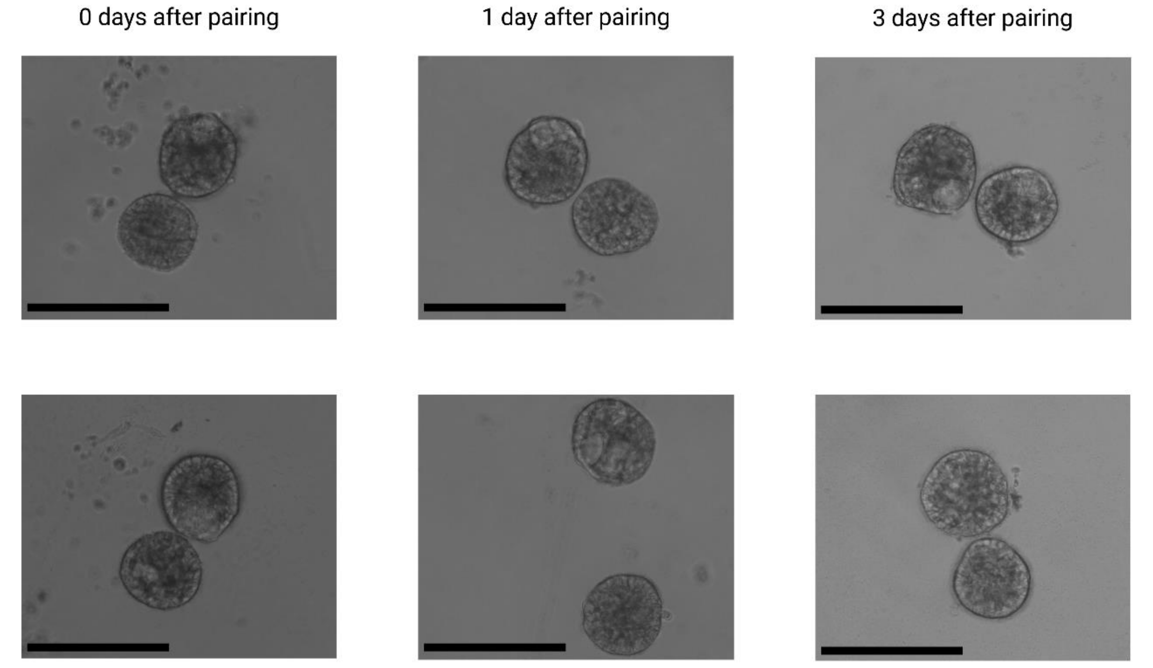


**Supplementary Figure S1. Inability of mature CiliaBots to aggregate.** Fully matured CiliaBots were brought into contact with one another on following 3 weeks of differentiation. Images were subsequently taken 1 and 3 days following initial contact, showcasing that mature CiliaBots appear to have no tendency to aggregate. Scale bar, 275 μm. Created in BioRender. Ren, X. (2025) https://BioRender.com/co4kvzb

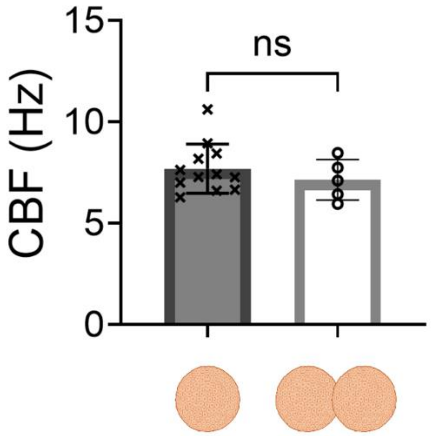


**Supplementary Figure S2. Ciliary beat frequencies (CBF) observed in AggreBots.** Measured CBF values in $CBB_1$ UniBots ($n$ = 12) and $CBB_2$ M1 AggreBots ($n$ = 5) via kymograph

analysis of collagen-embedded CiliaBots. Created in BioRender. Ren, X. (2025) https://BioRender.com/lotii2u

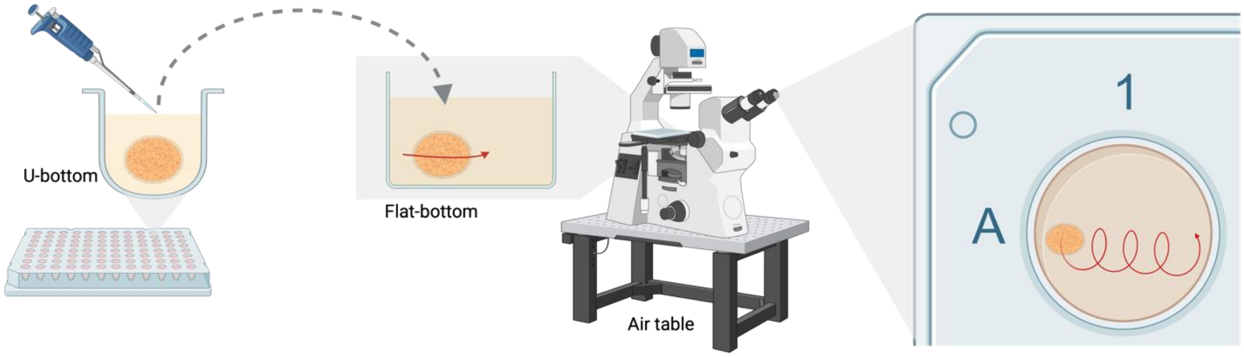


**Supplementary Figure S3. Schematic depiction of process behind characterization of CiliaBot motility.** Created in BioRender. Ren, X. (2025) https://BioRender.com/bzrr2ng

## Supplementary Tables

| | Donor 1 | | Donor 2 | |
|---|---|---|---|---|
| | Success | Failure | Success | Failure |
| M1 | 50 | 0 | 10 | 0 |
| M3 | 49 | 0 | 10 | 0 |
| M5 | 41 | 9 | 7 | 3 |
| Chi-square Result | $p < 0.001$ | | $p < 0.05$ | |

**Supplementary Table S1. Aggregation success rate as function of CBB age and cell donor.** Aggregation success or failure for each CBB pair, separated by age pre-contact (M1, M3, M5) from two cell donors. Donor 1 represents the cell donor utilized in all of the main figures of the paper. Chi-square test for trend for both donors.

| Cell Line | Age | Sex |
|---|---|---|
| Non-diseased (Donor 1) | 11 | M |
| Non-diseased (Donor 2) | 43 | M |

| | | |
|---|---|---|
| *CCDC39* gene-mutant c.830_831delCA (p.Thr277Argfs∗3) and c.1871_1872delTA (p.Ile624Lysfs∗3) | 57 | M |

**Supplementary Table S2. Donor information for utilized cells.** Age and sex information for cell lines used over the course of this study.

| Panel | Sample Count |
|---|---|
| Figure 2B | 0 hrs: *n* = 22, 2 hrs: *n* = 22, 4 hrs: *n* = 22, 6 hrs: *n* = 21, 24 hrs: *n* = 22 |
| Figure 2C | M1<br>t+0: *n* = 39, t+3: *n* = 39, Day 20: *n* = 28<br>M3<br>t+0: *n* = 38, t+3: *n* = 37, Day 20: *n* = 23<br>M5<br>t+0: *n* = 35, t+3: *n* = 33, Day 20: *n* = 21 |
| Figure 2D | M1: *n* = 10, M3: *n* = 7, M5: *n* = 6, UniBot: *n* = 7 |
| Figure 3D | UniBot<br>500-Cell: *n* = 18, 1000-Cell: *n* = 23, 1500-Cell: *n* = 21, 2000-Cell: *n* = 24<br>AggreBot<br>1000-Cell: *n* = 18, 1500-Cell: *n* = 19, 2000-Cell: *n* = 20 |
| Figure 3E | UniBot<br>500-Cell: *n* = 18, 1000-Cell: *n* = 22, 1500-Cell: *n* = 18, 2000-Cell: *n* = 21<br>AggreBot<br>1000-Cell: *n* = 18, 1500-Cell: *n* = 18, 2000-Cell: *n* = 20 |
| Figure 3F | UniBot<br>500-Cell: *n* = 18, 1000-Cell: *n* = 22, 1500-Cell: *n* = 18, 2000-Cell: *n* = 21<br>AggreBot<br>1000-Cell: *n* = 18, 1500-Cell: *n* = 18, 2000-Cell: *n* = 20 |
| Figure 3G | UniBot<br>500-Cell: *n* = 18, 1000-Cell: *n* = 22, 1500-Cell: *n* = 18, 2000-Cell: *n* = 21<br>AggreBot<br>1000-Cell: *n* = 18, 1500-Cell: *n* = 18, 2000-Cell: *n* = 20 |
| Figure 4A | ND: *n* = 10, PCD: *n* = 5 |
| Figure 4B | M1<br>t+0: *n* = 39, t+3: *n* = 37, Day 20: *n* = 22<br>M3<br>t+0: *n* = 35, t+3: *n* = 38, Day 20: *n* = 22<br>M5<br>t+0: *n* = 20, t+3: *n* = 5, Day 20: *n* = 2 |
| Figure 4D | Control: *n* = 19, XMU-MP-1: *n* = 12, NSC23766: *n* = 13,<br>XMU-MP-1+NSC23766: *n* = 12 |

| | |
|---|---|
| Figure 4G | $CBB_1^{ND}$-$CBB_1^{PCD}$: $n = 5$<br>$CBB_2^{ND}$-$CBB_1^{PCD}$: $n = 5$<br>$CBB_1^{ND}$-$CBB_2^{PCD}$: $n = 4$ |
| Figure 5B | $CBB_2^{ND}$ M1: $n = 30$<br>$CBB_2^{ND}$ M3: $n = 37$<br>$CBB_2^{ND}$ M5: $n = 31$<br>$CBB_1^{ND}$-$CBB_1^{PCD}$ M1: $n = 29$<br>$CBB_1^{ND}$-$CBB_1^{PCD}$ M3: $n = 37$<br>$CBB_1^{ND}$-$CBB_1^{PCD}$ M5: $n = 4$ |
| Figure 5C | $CBB_2^{ND}$ M1: $n = 30$<br>$CBB_2^{ND}$ M3: $n = 37$<br>$CBB_2^{ND}$ M5: $n = 31$<br>$CBB_1^{ND}$-$CBB_1^{PCD}$ M1: $n = 29$<br>$CBB_1^{ND}$-$CBB_1^{PCD}$ M3: $n = 34$<br>$CBB_1^{ND}$-$CBB_1^{PCD}$ M5: $n = 4$ |
| Figure 5D | $CBB_2^{ND}$ M1: $n = 30$<br>$CBB_2^{ND}$ M3: $n = 37$<br>$CBB_2^{ND}$ M5: $n = 31$<br>$CBB_1^{ND}$-$CBB_1^{PCD}$ M1: $n = 29$<br>$CBB_1^{ND}$-$CBB_1^{PCD}$ M3: $n = 34$<br>$CBB_1^{ND}$-$CBB_1^{PCD}$ M5: $n = 4$ |
| Figure 5E | $CBB_2^{ND}$ M1: $n = 30$<br>$CBB_2^{ND}$ M3: $n = 37$<br>$CBB_2^{ND}$ M5: $n = 31$<br>$CBB_1^{ND}$-$CBB_1^{PCD}$ M1: $n = 29$<br>$CBB_1^{ND}$-$CBB_1^{PCD}$ M3: $n = 34$<br>$CBB_1^{ND}$-$CBB_1^{PCD}$ M5: $n = 4$ |
| Figure 5F | $CBB_3^{ND}$: $n = 25$<br>$CBB_2^{ND}$-$CBB_1^{PCD}$: $n = 20$<br>$CBB_1^{ND}$-$CBB_2^{PCD}$: $n = 22$ |
| Figure 5G | $CBB_3^{ND}$: $n = 24$<br>$CBB_2^{ND}$-$CBB_1^{PCD}$: $n = 20$<br>$CBB_1^{ND}$-$CBB_2^{PCD}$: $n = 22$ |
| Figure 5H | $CBB_3^{ND}$: $n = 24$<br>$CBB_2^{ND}$-$CBB_1^{PCD}$: $n = 20$<br>$CBB_1^{ND}$-$CBB_2^{PCD}$: $n = 22$ |
| Figure 5I | $CBB_3^{ND}$: $n = 24$<br>$CBB_2^{ND}$-$CBB_1^{PCD}$: $n = 20$<br>$CBB_1^{ND}$-$CBB_2^{PCD}$: $n = 22$ |
| Figure 5J | $CBB_4^{ND}$: $n = 18$<br>$CBB_3^{ND}$-$CBB_1^{PCD}$: $n = 23$ |

| | |
|---|---|
| | Chiral $CBB_2^{ND}$-$CBB_2^{PCD}$: $n = 18$<br>Symmetric $CBB_2^{ND}$-$CBB_2^{PCD}$: $n = 33$ |
| Figure 5K | $CBB_4^{ND}$: $n = 18$<br>$CBB_3^{ND}$-$CBB_1^{PCD}$: $n = 23$<br>Chiral $CBB_2^{ND}$-$CBB_2^{PCD}$: $n = 18$<br>Symmetric $CBB_2^{ND}$-$CBB_2^{PCD}$: $n = 33$ |
| Figure 5L | $CBB_4^{ND}$: $n = 18$<br>$CBB_3^{ND}$-$CBB_1^{PCD}$: $n = 23$<br>Chiral $CBB_2^{ND}$-$CBB_2^{PCD}$: $n = 18$<br>Symmetric $CBB_2^{ND}$-$CBB_2^{PCD}$: $n = 33$ |
| Figure 5M | $CBB_4^{ND}$: $n = 18$<br>$CBB_3^{ND}$-$CBB_1^{PCD}$: $n = 23$<br>Chiral $CBB_2^{ND}$-$CBB_2^{PCD}$: $n = 18$<br>Symmetric $CBB_2^{ND}$-$CBB_2^{PCD}$: $n = 33$ |
| Figure 6A | Pheno-cluster 1: $n = 67$<br>Pheno-cluster 2: $n = 154$<br>Pheno-cluster 3: $n = 102$ |
| Figure 6B | Pheno-cluster 1: $n = 67$<br>Pheno-cluster 2: $n = 154$<br>Pheno-cluster 3: $n = 102$ |
| Figure 6C | Pheno-cluster 1: $n = 67$<br>Pheno-cluster 2: $n = 154$<br>Pheno-cluster 3: $n = 102$ |
| Figure 6D | Pheno-cluster 1: $n = 67$<br>Pheno-cluster 2: $n = 154$<br>Pheno-cluster 3: $n = 102$ |
| Figure 6E | $CBB_2^{ND}$: $n = 30$<br>$CBB_1^{ND}$-$CBB_1^{PCD}$: $n = 29$ |
| Figure 6F | $CBB_3^{ND}$: $n = 24$<br>$CBB_2^{ND}$-$CBB_1^{PCD}$: $n = 20$<br>$CBB_1^{ND}$-$CBB_2^{PCD}$: $n = 22$ |
| Figure 6G | $CBB_4^{ND}$: $n = 18$<br>$CBB_3^{ND}$-$CBB_1^{PCD}$: $n = 23$<br>Chiral $CBB_2^{ND}$-$CBB_2^{PCD}$: $n = 18$<br>Symmetric $CBB_2^{ND}$-$CBB_2^{PCD}$: $n = 33$ |

**Supplementary Table S3. Sample sizes for all experiments.** *n*-values for all experimental conditions in the main figures, separated by panel.

**Supplementary Movies**

**Supplementary Movie S1.** Exterior cilia agitate CiliaBots, preventing stable contact and aggregation. Scale bar 275 μm.

**Supplementary Movie S2.** 30X Speed video depicting locomotion of a $CBB_2^{ND}$ AggreBot, alongside visualization of the traced loop-de-loop via the "path-and-extent" method. Scale bar 1000 μm.

**Supplementary Movie S3.** Locomotion of a $CBB_3^{ND}$ AggreBot. Scale bar 1000 μm.

**Supplementary Movie S4.** Locomotion of a $CBB_4^{ND}$ AggreBot. Scale bar 1000 μm.

**Supplementary Movie S5.** Locomotion of a $CBB^{ND}$ UniBot. Scale bar 1000 μm.

**Supplementary Movie S6.** Lack of motility from a $CBB^{PCD}$ UniBot. Scale bar 1000 μm.

**Supplementary Movie S7.** Cilia activity of a collagen-embedded $CBB_1^{ND}$-$CBB_1^{PCD}$ AggreBot. Scale bar 75 μm.

**Supplementary Movie S8.** Cilia activity of a collagen-embedded $CBB_2^{ND}$-$CBB_1^{PCD}$ AggreBot. Scale bar 75 μm.

**Supplementary Movie S9.** Cilia activity of a collagen-embedded $CBB_1^{ND}$-$CBB_2^{PCD}$ AggreBot. Scale bar 75 μm.

**Supplementary Movie S10.** Locomotion of a $CBB_2^{ND}$ AggreBot. Scale bar 1000 μm.

**Supplementary Movie S11.** Locomotion of a $CBB_1^{ND}$-$CBB_1^{PCD}$ AggreBot, showcasing the decreased translational speed and increased path curvature brought about by the incorporation of $CBB^{PCD}$, with $CBB^{PCD}$ marked with red "extents." Scale bar 1000 μm.

**Supplementary Movie S12.** Locomotion of a $CBB_3^{ND}$ AggreBot. Scale bar 1000 μm.

**Supplementary Movie S13.** Locomotion of a $CBB_1^{ND}$-$CBB_2^{PCD}$ AggreBot. Scale bar 1000 μm.

**Supplementary Movie S14.** Locomotion of a $CBB_4^{ND}$ AggreBot. Scale bar 1000 μm.

**Supplementary Movie S15.** Locomotion of a chiral $CBB_2^{ND}$-$CBB_2^{PCD}$ AggreBot. Scale bar 1000 μm.

**Supplementary Movie S16.** Locomotion of a symmetric $CBB_2^{ND}$-$CBB_2^{PCD}$ AggreBot. Scale bar 1000 μm.

**Supplementary Movie S17.** Locomotion of an AggreBot with a high linear tendency. Scale bar 1000 μm.

**Supplementary Movie S18.** Locomotion of an AggreBot with a high linear tendency. Scale bar 1000 μm.

**Supplementary Movie S19.** Locomotion of an AggreBot with a high linear tendency. Scale bar 1000 µm.
